# Loss of Cav1.2 channels impairs hippocampal theta burst stimulation-induced long-term potentiation

**DOI:** 10.1080/19336950.2020.1807851

**Published:** 2020-08-15

**Authors:** Preethy S. Sridharan, Yuan Lu, Richard C Rice, Andrew A. Pieper, Anjali M. Rajadhyaksha

**Affiliations:** a Harrington Discovery Institute, University Hospitals Cleveland Medical Center, Cleveland, OH, USA; b Department of Psychiatry and Department of Neuroscience, Case Western Reserve University, Cleveland, OH, USA; c Department of Psychiatry, University of Iowa Carver College of Medicine, Iowa City, IA, USA; d Weill Cornell Autism Research Program, Weill Cornell Medicine of Cornell University, New York, NY, USA; e Pediatric Neurology, Pediatrics, Weill Cornell Medicine of Cornell University, New York, NY, USA; f Geriatric Psychiatry, GRECC, Louis Stokes Cleveland VA Medical Center, Cleveland, OH, USA; g Feil Family Brain and Mind and Research Institute, Weill Cornell Medicine of Cornell University, New York, NY, USA

**Keywords:** Calcium, Cav1.2, long-term potentiation

## Abstract

CACNA1 C, which codes for the Ca_v_1.2 isoform of L-type Ca^2+^ channels (LTCCs), is a prominent risk gene in neuropsychiatric and neurodegenerative conditions. A role forLTCCs, and Ca_v_1.2 in particular, in transcription-dependent late long-term potentiation (LTP) has long been known. Here, we report that elimination of Ca_v_1.2 channels in glutamatergic neurons also impairs theta burst stimulation (TBS)-induced LTP in the hippocampus, known to be transcription-independent and dependent on N-methyl D-aspartate receptors (NMDARs) and local protein synthesis at synapses. Our expansion of the established role of Ca_v_1.2channels in LTP broadens understanding of synaptic plasticity and identifies a new cellular phenotype for exploring treatment strategies for cognitive dysfunction.

## Introduction

The role of the Ca_v_1.2 isoform of L-type Ca^2+^ channels (LTCCs) is well known in hippocampal-mediated long-term memory and related behaviors [1–5].The clinical relevance of this relationship is bolstered by the fact that*CACNA1 C*, the gene that encodesCa_v_1.2, is a prominent risk gene for a wide array of neuropsychiatric [2,3,6,7]andneurodegenerative [8–11]disorders that manifest with cognitive impairment.

Hippocampal LTP at CA3 Schaffer collaterals to CA1 neurons is a common synaptic model of learning and memory [12,13], and it has long been known that Ca^2+^ influx from LTCCs plays an important role in N-methyl-D-aspartate receptor (NMDAR)-independent, transcription-dependent LTP [14–18]. This is also consistent with the critical role of LTCCs in activity-dependent transcription and long-term memory [19–22]. Recently, we have reported that Ca_v_1.2 channels additionally regulate local synaptic protein synthesis through adjusting mTORC1protein translational machinery [23]. Since this same mechanism is also involved in NMDAR-dependent, transcription-independent LTP [24], we hypothesized that Ca_v_1.2 channels might be required for this earlier form of LTP as well. NMDAR-dependent, transcription-independent LTP can be elicited by theta burst stimulation (TBS) at glutamatergic synapses in the hippocampus [25–27], and TBS closely mimics the natural rhythms of neuronal activity in the brain [25,28]. Importantly, TBS delivery through transcranial magnetic stimulation has been approved by the United States Food and Drug Administration for the treatment of major depression [29,30]. Thus, we examined whether conditional deletion of Ca_v_1.2 in forebrain glutamatergic neurons (Ca_v_1.2^KO^) would disrupt TBS-induced LTP at Schaffer collateral/CA1 synapses.

## Materials and methods

### Animals

All animal procedures were performed in accordance with the policies and regulations of the University of Iowa and Weill Cornell Medicine institutional animal care and use committees. Male CamK2-Cre Ca_v_1.2^KO^ mice and wild type (WT) littermates maintained on a C57Bl/6 J background were used. Mice were housed in temperature-controlled conditions, provided food and water *ad libitum*, and maintained on a 12-h light/dark cycle.

### Preparation of acute hippocampal slices

Sagittalhippocampal slices (400 μm) from adult mice (> postnatal day 60) were cut using a Vibratome 1000 Plus (Vibratome, St. Louis, MO) in ice-cold slicing buffer (in mM: 127 NaCl, 26 NaHCO3, 1.2 KH2PO4, 1.9 KCl, 1.1 CaCl2, 2 MgSO4, 10 D-Glucose) bubbled with 95% O2 and 5% CO2. Slices were transferred to a holding chamber containing oxygenated artificial cerebrospinal fluid (ACSF; in mM: 127 NaCl, 26 NaHCO3, 1.2 KH2PO4, 1.9 KCl, 2.2 CaCl2, 1 MgSO4, 10 D-Glucose) for 30 min at 34°C and for another 30 min at 22°C for recovery. Slices were then transferred to a submersion recording chamber continually perfused with 32°C oxygenated artificial cerebrospinal fluid (ACSF) (rate: 2 ml/min). Slices were equilibrated for at least 15 min before each recording.

### Electrophysiology

ACSF-filled glass electrodes (resistance <1 MΩ) were positioned in the stratum radiatum of area CA1 for extracellular recording. Synaptic responses were evoked by stimulating Schaffer collaterals with 0.2 ms pulses once every 15 s. The stimulation intensity was systematically increased to determine the maximal field excitatory post-synaptic potential (fEPSP) slope and then adjusted to yield 40–60% of the maximal (fEPSP) slope. Experiments with maximal fEPSPs of less than 0.5 mV, with large fiber volleys, or with substantial changes in the fiber volley during recording, were rejected. LTP was induced by 12TBS (12 bursts, each of 4 pulses at 100 Hz, with pulse duration of 0.2 ms and 5Hzinterburst frequency). Field EPSPs were recorded (AxoClamp 900A amplifier, Axon Instruments, Foster City, CA), filtered at 1 kHz, digitized at 10 kHz (Axon Digidata 1440), and stored for off-line analysis (Clampfit 10). Initial slopes of fEPSPs were expressed as percentages of baseline averages. In the summary graph of LTP, each point represents the average of four consecutive responses. Time-matched, normalized data were averaged across experiments and expressed as means±SEM.

### Subcellular fractionation and immunoblotting

Synaptosomal fractions from adult (> P60) hippocampus were generated as previously described [23,31]and used for western blot analysis. Briefly, tissue was homogenized in 0.3 M sucrose/0.01 mM HEPES buffer containing protease and phosphatase inhibitors and centrifuged at 1000xg. The supernatant was then spun again at1000xg, with the subsequently obtained fresh supernatant spun at 12,000xg. The final pellet was resuspended in 4 mM HEPES/1 mM EDTA buffer and used as the synaptosome fraction. Protein concentrations were determined using the BCA assay, and protein lysates were separated on a 10% SDS gel along with a Kaleidoscope-prestained protein standard (Bio-Rad, Hercules, CA). Blots were blocked in 5% nonfat dry milk for 1 h and incubated in primary antibody (Table 1) for 12–48 h on a shaker at 4°C. Incubation in secondary antibody was performed at room temperature for 1 h in horseradish peroxidase-linked IgG conjugated antibody. Membranes were visualized using Western Lightning Chemiluminescence solution (Perkin Elmer Life Science, Boston, MA) and optical density was analyzed using NIHImage (NIH, Bethesda, MD). Immunoblot data were analyzed using an independent samples t-test, performed by Prism 8 Graphpad software. Proteins were normalized to GAPDH, which was used as a loading control. Western analyses were done using X-ray film.

**Table 1.** List of antibodies used for immunoblots.

Protein	Company	Catalog number	RRID	Antibody concentration	Molecular Weight (kDa)
GluN1	Millipore	Ab9864	10,807,557	1:1000	120
GluN2A	NeuroMab	75–288	2,307,331	1:1000	170
GluN2B	Millipore	06–600	310,193	1:1000	180
mTOR	Cell Signaling Technology	2972	330,978	1:1000	250
p-mTOR S2448	Cell Signaling Technology	2971	330,970	1:1000	250
GAPDH	Abcam	Ab22555	447,153	1:10,000	36

### Statistics

Electrophysiological data were time-matched, normalized, and averaged across experiments and expressed as mean±SEM. LTP was analyzed using a two-tailed unpaired t-test and significant differences were determined as a p value <0.05. Immunoblot data were analyzed using an independent samples t-test. Proteins were normalized to GAPDH, which was used as a loading control. All statistical analysis was performed by Prism 8 Graphpad software.

## Results

**Figure 1.**
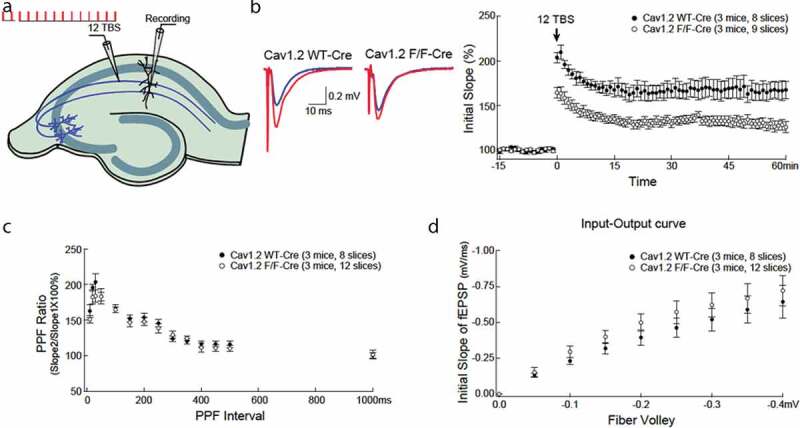
Long-term potentiation (LTP) is significantly impaired in male CamK2Cre, Ca_v_1.2^KO^ mice without apparent alteration in paired-pulse facilitation or input-output curve. (a) Illustration of the recording scheme. The Schaffer collateral pathway projecting to CA1 neurons was stimulated with bipolar stimulating electrodes. LTP was induced by theta burst stimulation (12TBS; 12 bursts, each of 4 pulses at 100 Hz). (b) Example traces before (blue) and after (red) TBS. LTP (at time 60 min) is substantially reduced in CamK2Cre, Ca_v_1.2^KO^ mice compared to wildtype (WT) littermate controls (166 ± 10% vs. 128 ± 6%, t_(15)_ = 3.549, p = 0.0029). Post-stimulation potentiation (at time 0 min) was also significantly reduced in CamK2Cre, Ca_v_1.2^KO^ mice (180 ± 7% vs. 145 ± 5%, t_(15)_ = 4.220, p = 0.0007). (c)There was no difference in paired pulse facilitation between CamK2Cre, Ca_v_1.2^KO^ mice and WT littermate controls over a wide range of inter-stimulus intervals, indicating intact presynaptic machinery in CamK2Cre, Ca_v_1.2^KO^ mice. (d) Input-output curves with the postsynaptic response (initial slope of field excitatory postsynaptic potential (fEPSP)) plotted as a function of the presynaptic fiber volley amplitude were indistinguishable between CamK2Cre, Ca_v_1.2^KO^ mice and WT mice, indicating intact baseline synaptic transmission.

We determined the effect of forebrain-specific deficiency of Cav1.2 onTBS-induced LTP in adult Ca_v_1.2^KO^ mice and wild type littermates. Recordings were obtained for 60 minutes. Stimulating pulses were delivered to the Schaffer collateral fibers projecting from CA3 to CA1 pyramidal cells, with recording electrodes placed in the CA1 region of the hippocampus (Figure 1(a)). LTP was quantified as the field excitatory postsynaptic potential (fEPSP) slope as a percentage of baseline.Ca_v_1.2^KO^micedemonstrated significantly reduced LTP immediately after TBS, and the reduction was sustained for 60 minutes (Figure 1(b)). Measurement of paired-pulse facilitation, an index of presynaptic probability of release, showed that impaired TBS-induced LTP was independent of altered presynaptic machinery, as there was no difference across a wide range of inter-stimulus intervals between Ca_v_1.2^KO^and WT littermate controls (Figure 1(c)). In addition, no difference in baseline synaptic transmission was observed, based on input/output curves from Ca_v_1.2^KO^and WT littermate controls (Figure 1(d)).

**Figure 2.**
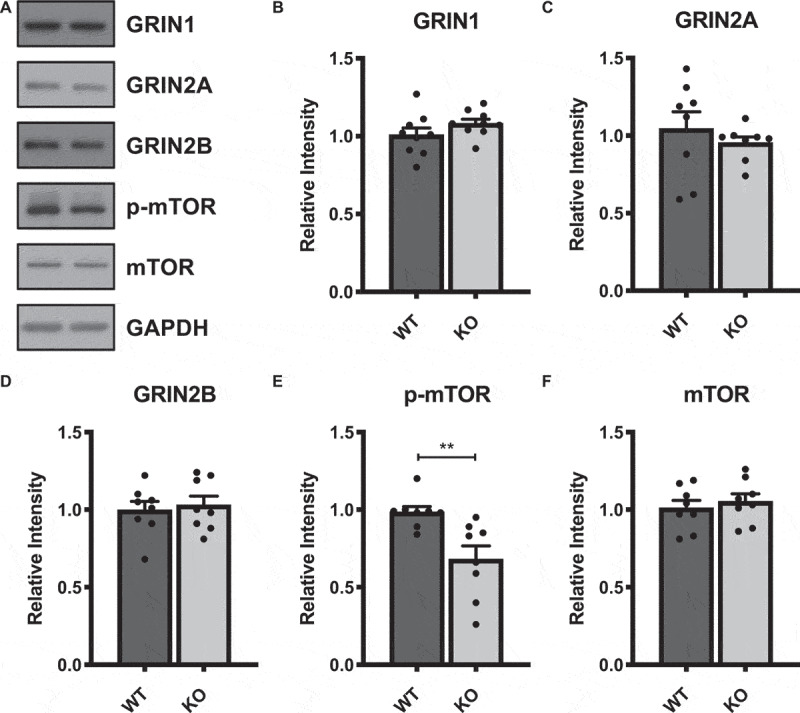
Western blots from isolated synaptosomal fractions of dorsal hippocampus of CamK2Cre, Ca_v_1.2^KO^ mice and WT littermates reveal a decrease on phosphorylated mTOR expression. (a) Representative bands of NMDAR subunits (GRIN1, GRIN2A, and GRIN2B), S2448 phosphorylated mTOR (p-mTOR), and total mTOR protein levels taken from the same blot and adjacent lanes. (b-f) Quantification of relative intensities of respective bands, normalized to GAPDH expression levels.P-mTOR, t_(14)_ = 3.201**p = 0.0064. Data are displayed as mean ± SEM.

Given that TBS-induced LTP is NMDAR- and mTORC1-dependent [24,27], we next questioned whether the impaired TBS-induced LTP in Ca_v_1.2^KO^mice might be related to altered levels of NMDAR subunits and phosphorylated mTOR at serine 2448, a marker of active mTORC1 [32].Western blots using synaptosomal fractions of dorsal hippocampus (Figure 2(a)) revealed no difference in levels of NMDAR subunitsGRIN1, GRIN2A, or GRIN2B (Figure 2(b-d)), but did show lower levels of S2448phospho-mTOR (Figure 2(e)), without any difference in total mTOR protein (Figure 2(f)).

## Discussion

Until now, LTCCs in neurons have been predominantly studied in the context of their requisite role in induction of NMDAR-independent, transcription-dependent LTP [14–18,33]. With the data presented here, however, we have now established an additional role for Ca_v_1.2 in TBS-induced LTP, which is known to be transcription-independent and dependent on N-methyl D-aspartate receptors (NMDARs) and local protein synthesis at synapses [24]. Specifically, we observed that mice lacking Ca_v_1.2 channels in glutamatergic neurons are impaired inTBS-induced LTPat Schaffer collateral to CA1 synapses, while basal synaptic transmission and presynaptic function are intact. We find no change in NMDAR subunit (GRIN1, GRIN2A, or GRIN2B) levels in hippocampal synaptosomal fractions (generated from the entire hippocampus) as a consequence of Ca_v_1.2 knockout, but do observe reduced levels of active mTORC1, a marker for local protein synthesis. This is reflected by a small decrease in phosphorylated mTOR. It is important to note, however, that these associative results do not prove a causal relationship to diminished TBS-induced LTP. Future studies will address this question at the Schaffer collateral/CA1 synapse. It is also important to note that deletingCa_v_1.2 could conceivably affect NMDAR activity without affecting overall expression levels of NMDAR subunits. Future work to directly measure NMDAR currents will be required to definitively address this possibility. Further investigation will also be necessary to parse the specific mechanisms that relate to our findings. There are several well-studied pathways downstream of Ca_v_1.2 channels that regulate LTP, such as BDNF signaling [34–36], which can be locally translated at the synapse to contribute to synaptic plasticity. Likewise, the CaM Kinase II pathway, which is activated by Ca_v_1.2 [37,38] and enriched in dendritic spines during LTP [39], could result in increased α-amino-3-hydroxy-5-methyl-4-isoxazolepropionic acid receptor (AMPAR) conductance at synapses via CaMKII-mediated phosphorylation of AMPAR subunits, a key mechanism for induction of LTP and synaptic plasticity [40]. In conclusion, our data show that Ca_v_1.2 is required forTBS-induced LTP, which may depend on local synaptic mechanisms in the adult hippocampus.

At first glance, our results appear to contradict a previous report of no role for Ca_v_1.2 in this form of LTP [17]. This difference is likely due to the different promoters that were used to drive Cre recombinase expression during the creation of the two different strains of Ca_v_1.2^KO^ mice. In the previous report, Moosmang et al. [17]used the*Nex* promoter, which is activated during development at embryonic day 12 [41]. By contrast, here we used the*alpha-CamK2* promoter, which is not activated until postnatal day 18 [42]. Since Ca_v_1.2calcium signaling is a critical regulator of early neuronal, dendritic and synaptic development [43–47], very early elimination of Ca_v_1.2 via the*Nex*promoter could lead to developmental adaptations that might allow sufficient synaptic strengthening for the maturation of the embryonic brain necessary for viability. This adaptation could then result in an adult brain deficient in Ca_v_1.2that is still able to execute TBS-induced LTP. Our results here, in which Ca_v_1.2has been selectively eliminated at a much later date (~postnatal day 21), likely represent more faithfully the role of Ca_v_1.2channels in the adult brain. In addition, a role of Ca_v_1.2 channels in TBS-induced LTP is compatible with previous reports of Ca_v_1.2 channel-mediated NMDAR-signaling [48,49] as well as our recent discovery that loss of Ca_v_1.2 results in decreased activation of mTORC1 [23], which is required for TBS-induced LTP [24].Future identification of the molecular adaptations in the *Nex* promoter-driven versus *CaMK2* promoter-driven Ca_v_1.2 KO mouse models could provide insight into early versus later Ca_v_1.2neurodevelopmental processes.

We also note that a critical role for Ca_v_1.2in TBS-induced LTP could be related to the neurocognitive deficits that we and others have previously observed in these same mice [23,50–52], and this form of LTP could result from Ca_v_1.2-mediated hippocampal phenotypes [35,50]. Interestingly, this impairment in LTP is reminiscent of our previously reported findings in the methyl-CpG binding protein 2 (MECP2)-deficient mouse model of Rett syndrome [53], and MECP2 is a downstream target of LTCCs [54]. Conceivably, deficits in TBS-induced LTP may represent a commonality across neuropsychiatric disorders with dysregulated local protein synthesis and cognitive deficits [55,56].In conclusion, this previously unknown role of Ca_v_1.2 in TBS-induced LTPprovidesnew direction for studying Ca_v_1.2 channel mechanisms in this form of LTP and developing potential therapeutics in neuropsychiatric and neurodegenerative disease.
